# Supplementary material for: Empirical methods for controlling false positives and estimating confidence in ChIP-Seq peaks
Source: BMC Bioinformatics. 2008 Dec 5;9:523. doi: 10.1186/1471-2105-9-523 (PMC2628906; doi:10.1186/1471-2105-9-523)
Supplement: Additional File 1 — A variety of html documents from the USeq web site detailing the available applications, their best usage, output file type descriptions, command line menus, etc. [file 1471-2105-9-523-S1.zip › USeqUserGuides/JavaDoc.zip!/JavaDoc/gata/geneGlyphs/TransGrpGlyph.html]

TransGrpGlyph


|  |  |  |
| --- | --- | --- |
| **Overview** \| **Package** \| **Class** \| **Use** \| **Tree** \| **Deprecated** \| **Index** \| **Help** | |  |
| **PREV CLASS**   NEXT CLASS | **FRAMES**    **NO FRAMES**     **All Classes** |  |
| SUMMARY: NESTED \| FIELD \| CONSTR \| METHOD | DETAIL: FIELD \| CONSTR \| METHOD |  |


---


## gata.geneGlyphs Class TransGrpGlyph

```
java.lang.Object
  gata.geneGlyphs.TransGrpGlyph
```

---

``` public class TransGrpGlyph extends java.lang.Object ```

**Author:**
:   Nix
    Container class holding components that make up a TransGroup (shapes for DNA, exons, prot....)

---

**Constructor Summary**

`TransGrpGlyph(TransGroup transGrp,
GATAParams gataParams,
AnnoSpecParams annoParams)`


| **Method Summary** | |
| --- | --- |
| `void` | `draw(double yCoord)` |
| `double` | `getYTop()`             Top most y coordinate for this transGroup, including transGrp spacer, start next Shape here for no overlap |

**Methods inherited from class java.lang.Object**

`equals, getClass, hashCode, notify, notifyAll, toString, wait, wait, wait`

**Constructor Detail**

### TransGrpGlyph

```
public TransGrpGlyph(TransGroup transGrp,
                     GATAParams gataParams,
                     AnnoSpecParams annoParams)
```


**Method Detail**

### draw

```
public void draw(double yCoord)
```

---


### getYTop

```
public double getYTop()
```

:   Top most y coordinate for this transGroup, including transGrp spacer, start
    next Shape here for no overlap


---


|  |  |  |
| --- | --- | --- |
| **Overview** \| **Package** \| **Class** \| **Use** \| **Tree** \| **Deprecated** \| **Index** \| **Help** | |  |
| **PREV CLASS**   NEXT CLASS | **FRAMES**    **NO FRAMES**     **All Classes** |  |
| SUMMARY: NESTED \| FIELD \| CONSTR \| METHOD | DETAIL: FIELD \| CONSTR \| METHOD |  |


---
